# The Effects of a Cerebellar Transcranial Direct-Current Stimulation-Based Preventive Exercise Program on Physical Function and Fall Reduction Efficacy in Community-Dwelling Older Adults

**DOI:** 10.3390/healthcare14020241

**Published:** 2026-01-18

**Authors:** Deone Kang, JongEun Yim

**Affiliations:** Department of Physical Therapy, Graduate School, Sahmyook University, 815, Hwarang-ro, Nowon-gu, Seoul 01795, Republic of Korea; ptkang09@naver.com

**Keywords:** transcranial direct-current stimulation, cerebellum, neural plasticity, aged

## Abstract

**Highlights:**

**What are the main findings?**

- Administration of cerebellar transcranial direct-current stimulation (c-tDCS) prior to an Otago Exercise Program (OEP)-based fall prevention exercise program significantly improved lower-extremity muscle strength (Five Times Sit to Stand Test, FTSST) and dynamic balance (Time Up to Go, TUG) in older adults.
- No significant changes were observed in static balance (Balancia) or fall efficacy (Falls Efficacy Scale—Korean ver., FES-K) following the short-term, 4-week intervention.

**What are the implications of the main findings?**

- c-tDCS may serve as an effective adjunct modality that enhances neuroplasticity and motor control, thereby amplifying the strength and dynamic balance benefits of fall prevention exercise programs.
- Longer intervention durations or multicomponent approaches may be required to improve static balance and reduce psychological fear of falling.

**Abstract:**

**Background/Objectives**: Falls are a major cause of injury in older adults, closely related to declines in muscle strength, balance control, and sensory integration. Although exercise-based fall prevention programs are well supported, evidence on combining such programs with cerebellar transcranial direct-current stimulation (c-tDCS) remains limited. This study investigated the effects of c-tDCS applied before a modified Otago Exercise Program (OEP) on lower-extremity strength, balance, and fall efficacy in older adults. **Methods**: In this randomized controlled study, twenty-six community-dwelling older adults (median age [IQR]: experimental, 74.00 [10] years; control, 71.00 [10] years) were randomly assigned to either a c-tDCS + exercise group (n = 13) or a sham + exercise group (n = 13). The intervention was administered twice weekly for four weeks. The experimental group received 15 min of c-tDCS followed by 30 min of OEP-based exercise; the control group received sham stimulation under identical conditions. The outcome measures included the Five Times Sit to Stand Test (FTSST), Timed Up and Go (TUG), Balancia-based static balance (velocity average), and Falls Efficacy Scale—Korea (FES-K). Assessments were performed pre- and post-intervention. **Results**: The experimental group demonstrated significantly greater improvements than the control group (*p* < 0.05) in the Five Times Sit to Stand Test (r = 0.44) and Timed Up and Go test (r = 0.56). No significant changes were observed in static balance or fall efficacy in either group (*p* > 0.05). **Conclusions**: The combined use of c-tDCS and an OEP-based fall prevention exercise program effectively improved lower-extremity strength and dynamic balance in older adults. However, short-term intervention did not influence static balance or fall efficacy. Further studies using longer intervention periods and larger samples are warranted to verify these findings and clarify the mechanisms underlying c-tDCS-enhanced motor performance.

## 1. Introduction

The global population aging is increasing rapidly, and estimates indicate that by 2030, one in six individuals worldwide will be aged 60 years or older [1]. In South Korea, the proportion of individuals aged 65 years and above is projected to reach 19.2% in 2024 and exceed 20% in 2025, classifying the nation as a “superaged society” [2]. The World Health Organization (WHO) has cautioned that this demographic shift will have profound implications for social structures, public health, and the global economy. Similarly, the United Nations [1] projected that the number of people aged 60 years and older will reach approximately 2.1 billion by 2050, emphasizing the urgent need to restructure the healthcare system to address the challenges posed by population aging.

Aging is accompanied by a progressive decline in the neuromuscular system, characterized by muscle fiber atrophy, loss of motor units, and reduced sensory integration capacity [3,4,5,6]. While sarcopenia, defined as the age-related loss of muscle mass, is a well-known risk factor for physical frailty, recent evidence suggests that the decline in muscle strength, termed dynapenia, often occurs more rapidly than the loss of muscle mass [7,8]. This dissociation implies that neurological factors, such as reduced central activation and impaired corticospinal excitability, play a critical role in the functional decline observed in older adults [8]. In particular, weakening of the lower-extremity musculature contributes to reduced weight bearing and propulsive capacity, leading to impairments in gait stability and postural control, and increasing the risk of falls [9]. Furthermore, aging is associated with declines in balance maintenance and walking speed, as well as increased gait variability, which are key predictors of fall risk [10]. Postural control relies on the integration of sensory input from the somatosensory, visual, and vestibular systems, which has also been shown to deteriorate with advancing age [3,5].

According to the World Health Organization (WHO) [11], approximately 28–35% of community-dwelling older adults aged 65 years and above experience at least one fall annually, and falls are identified as the leading cause of trauma in this population. Falls frequently result in fractures, musculoskeletal injuries, and head trauma, as well as psychological distress, reduced activity levels, loss of independence, and increased mortality [12,13]. Sherrington et al. [14] reported that balance-focused exercise programs can reduce fall rates by an average of 21%, while Liu-Ambrose et al. [15] demonstrated that combining resistance and agility training can decrease fall risk scores by more than 50%. Furthermore, the WHO emphasizes that a multifactorial exercise approach incorporating strength, balance, and sensory integration training is the most effective strategy for fall prevention in older adults.

Transcranial direct-current stimulation (tDCS) is a noninvasive neuromodulatory technique that alters neural excitability and inhibition at the stimulation site by applying a low-intensity direct current to the scalp [16]. This intervention not only modulates cortical excitability but also influences the functionally connected brain regions [17]. tDCS has been utilized to improve postural control and balance in older adults and has been shown to significantly enhance balance performance, as reflected by improvements in Berg Balance Scale scores [18]. Repeated sessions of anodal tDCS have been reported to augment the effects of postural training in older adults at high risk of falls, thereby improving balance and postural stability [19]. The cerebellum plays a critical role in postural control and motor learning, and cerebellar tDCS (c-tDCS) has been identified as a potential noninvasive intervention capable of enhancing balance function by modulating neural plasticity [20]. Moreover, high-definition c-tDCS has been shown to significantly reduce step length variability during dual-task gait in older adults [21]. Collectively, these findings suggest that c-tDCS may be beneficial for fall prevention in older adults.

The Otago Exercise Program (OEP) is an evidence-based fall prevention exercise program designed to enhance lower-extremity strength, balance, and gait function in older adults. Randomized controlled trials and meta analyses have demonstrated that OEP participation significantly reduces the incidence of falls and fall-related injuries [22]. This program promotes neuromuscular adaptation through progressive resistance training and improves sensory integration through balance exercises, thereby increasing postural control, reaction times, and fall efficacy [23]. According to Mgbeojedo [24], the OEP has demonstrated benefits for both physical and psychosocial functioning among community-dwelling and institutionalized older adults. Specifically, the program has been reported to reduce fall risk by improving overall physical performance, including lower-extremity muscle strength, balance, and walking ability. Furthermore, the OEP enhances objective measures of balance performance and perceived balanced confidence. Recent studies have increasingly focused on optimizing session structures to further improve muscle strength.

Elastic band exercise has reported benefits for muscle strength and functional mobility in older women, demonstrating significantly improved grip strength and performance on the 30 s chair stand test [25]. Similarly, Gargallo et al. [26] found that exercise interventions incorporating elastic bands positively influence physical function in older women. In addition, resistance band exercise has been recognized as a complementary intervention that may reduce frailty and alleviate depressive symptoms. However, some studies have reported no significant improvements in specific strength measures, such as grip strength or lower-extremity muscle strength, or activities of daily living [27].

Several studies have demonstrated that c-tDCS and the OEP can effectively improve postural balance and control and prevent falls in older adults and clinical populations [22,28]. Consequently, OEP-based exercise programs have been widely implemented as therapeutic interventions for fall prevention among older adults in community and clinical settings [22]. The rationale for combining c-tDCS with the OEP lies in the potential for priming the motor system to maximize the benefits of physical training. While the OEP improves function through repetitive practice, recent evidence suggests that c-tDCS can enhance the responsiveness of the motor system by inducing a state of heightened neuroplasticity, thereby facilitating the acquisition and consolidation of motor skills [29,30]. Specifically, in this study, the stimulation electrode was placed at the midline (point O) to target the cerebellar vermis. Unlike direct motor cortex stimulation, modulating the vermis influences the dentato-thalamo-cortical (DTC) pathway, which scales movement amplitude and regulates the reticulospinal tracts responsible for proximal muscle control and trunk stability [31,32]. By enhancing excitability in this specific region, we hypothesized that c-tDCS would not only refine balance but also improve the neural efficiency of motor unit recruitment in lower-extremity muscles during the OEP, offering a synergistic benefit over exercise alone [21,33,34].

However, evidence regarding the efficacy of fall prevention exercise programs that incorporate tDCS remains limited [18]. Therefore, the present study hypothesized that this combined intervention—priming the cerebellum prior to OEP—would significantly enhance balance performance, lower-extremity muscle strength, and fall-related self-efficacy compared to exercise alone [28].

Therefore, this study aimed to investigate the effects of a combined intervention involving cerebellar activation using tDCS and an OEP-based fall prevention exercise program on physical function and fall efficacy in older adults.

## 2. Materials and Methods

### 2.1. Ethical Statement

The study design was approved by the Sahmyook University Institutional Review Board (SYU 2025-08-022-001; approved on 19 September 2025). In accordance with the Declaration of Helsinki, all subjects were informed about the purpose and procedure of the study and gave their informed consent to participate in the study. The study protocol was retrospectively registered on the Open Science Framework (OSF) to ensure transparency (Registration DOI: https://osf.io/xywbv, accessed on 30 December 2025). All procedures were conducted in strict accordance with the approved protocol.

### 2.2. Participants

The participants in this study were older adults attending the Pyeongtaek Southern Senior Welfare Center in Pyeongtaek-si, Gyeonggi-do, who met the inclusion criteria and voluntarily agreed to participate. The required sample size was determined a priori using G*Power software (version 3.1.9.7) [35]. We referred to a systematic review and meta-analysis by Guo et al. [18], which reported significant effects of tDCS on balance control in older adults. To ensure sufficient statistical power, we conservatively estimated an effect size (*f*) of 0.32, with a significance level (α) of 0.05 and a power of 0.80. This calculation indicated a minimum of 11 participants per group (total N = 22). Accounting for a potential 20% dropout rate in the older adult population, we recruited a total of 26 participants (13 per group). Ultimately, 24 participants completed the study. Participants were randomly allocated to the experimental group (c-tDCS + fall prevention exercise program) or the control group (sham c-tDCS + fall prevention exercise program) using a computer-generated random number table. Group assignments were placed in sequentially numbered, opaque, sealed envelopes prepared by an independent researcher who was not involved in recruitment, intervention delivery, or outcome assessments. After baseline assessments were completed, group allocation was determined by opening the next envelope in sequence.

Participants and outcome assessors were blinded to group allocation throughout the study period. The care providers delivering the stimulation (active or sham) and the exercise sessions were not blinded; however, they were not involved in outcome assessments, and all sessions were delivered using a standardized protocol. Active and sham sessions followed identical electrode placement and procedural setup; in the sham condition, stimulation was applied for 30 s and then turned off for the remainder of the stimulation period.

The inclusion criteria were as follows: (1) individuals aged 65 years or older; (2) a score of 24 or higher on the Korean Mini-Mental State Examination (K-MMSE), which was selected to exclude dementia and ensure participants possessed sufficient cognitive ability to understand and follow the intervention instructions [36]; and (3) a score of 120 or higher on the Functional Independence Measure (FIM). The exclusion criteria were as follows: (1) moderate or severe limitations in exercise capacity due to cardiovascular, neurological, or musculoskeletal disorders; (2) a history of lower-extremity surgery or fracture within the previous six months; and (3) any contraindications for tDCS, such as the presence of metallic implants in the cranium or a history of seizures.

**Figure 1:**
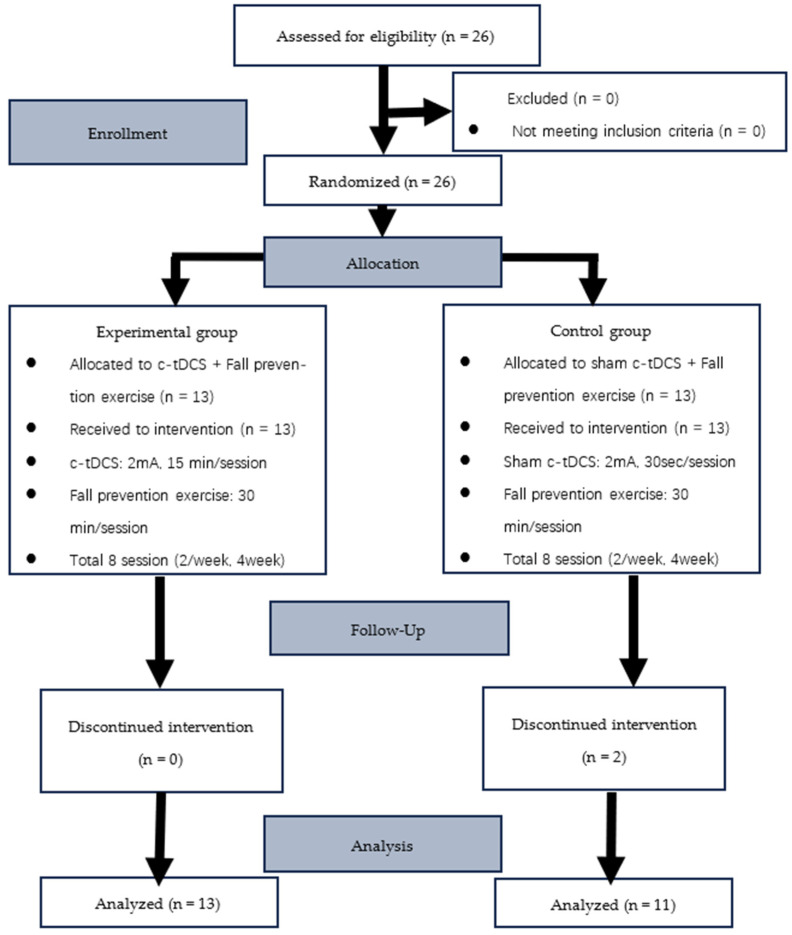
CONSORT flow diagram of participant enrollment, allocation, intervention, and analysis.

Participants were required to be capable of independently performing activities of daily living and ambulation without assistive devices and have no functional impairments in the visual, auditory, or vestibular systems. The flow of participant enrollment, allocation, intervention, and analysis is presented in Figure 1.

### 2.3. Procedure

Before the intervention, all participants underwent baseline assessments, including body composition analysis using InBody and questionnaire evaluations at the Pyeongtaek Southern Senior Welfare Center. Participants’ demographic and physical characteristics, including sex, age, height, and weight, were directly measured and recorded.

Physical function was evaluated before the intervention. Static balance was assessed using Balancia, with velocity average (Velavg, cm/s) as the outcome parameter. Dynamic balance was evaluated using the Timed Up and Go (TUG) test, and lower-extremity muscle strength was measured using the Five Times Sit to Stand Test (FTSST). Fall efficacy was measured using the Korean version of the Falls Efficacy Scale (FES-K).

The intervention was conducted twice weekly for four weeks. The experimental group received c-tDCS for 15 min, followed by a 30 min fall prevention exercise program, totaling 45 min per session for eight sessions. The control group received sham c-tDCS, in which stimulation was applied for 30 s and then turned off for the remaining 15 min, followed by the same 30 min fall prevention exercise program, also totaling 45 min per session for eight sessions.

To minimize the influence of extraneous variables, all participants were instructed to maintain their habitual daily physical activity levels and dietary patterns throughout the 4-week intervention period. Furthermore, they were asked to refrain from initiating any new exercise regimens or taking nutritional supplements that could affect muscle metabolism during the study.

Post intervention assessments were performed using the same measurement tools, Velavg (cm/s), TUG, FTSST, and the FES scale, to evaluate the treatment effects in both groups. Three licensed physical therapists, who were blinded to group allocation and were not involved in delivering the interventions, performed all measurements and data recordings.

### 2.4. Intervention

#### 2.4.1. Cerebellum Transcranial Direct-Current Stimulation (c-tDCS)

In this study, tDCS was applied to facilitate neuroplasticity within the cerebellum, thereby enhancing motor control and balance ability. c-tDCS was administered using a two channel direct-current stimulator (The Brain Driver v2.1, TheBrainDriver Corp., San Francisco, CA, USA). Following the protocol described in previous studies, the anodal electrode was positioned over the cerebellar region at point O according to the international 10–20 EEG system while the participant was seated. The cathodal electrode was placed over the left supraorbital area (FP1) (Figure 1). Stimulation was delivered at an intensity of 2 mA for 15 min [33,37]

#### 2.4.2. Modified Otago Exercise Program—Fall Prevention Exercise Program

The fall prevention exercise program was developed by adapting the framework of the Otago Exercise Program (OEP) to the physical characteristics of the participants [38]. The program was administered twice weekly for 4 weeks, with a progressive increase in exercise difficulty over the intervention period. The four-week intervention duration was selected to specifically target the early phase of neural adaptation. Previous studies indicate that initial improvements in muscle strength and motor control during the first few weeks of training are primarily driven by neural mechanisms—such as increased motor unit recruitment and firing rates—rather than structural muscle hypertrophy [39,40]. Furthermore, tDCS has been shown to accelerate motor learning and consolidation, potentially eliciting functional improvements within shorter timeframes compared to exercise alone [29,41]. Therefore, this study aimed to verify whether the cumulative effect of c-tDCS over a four-week period could sufficiently induce these neurally mediated functional gains. Each session lasted 30 min and consisted of 5 min of warm-up exercises (deep breathing, head movements, and neck movements), 10 min of muscle strengthening exercises (calf raises, toe raises, trunk movements, knee bends, and hip abduction), 10 min of balance training (standing still, sideways walking, backward walking, tandem stance, tandem walking, one-leg stance, and sit to stand), and 5 min of cool-down exercises (trunk extension and deep breathing).

#### 2.4.3. Elastic Band

In this study, elastic bands were incorporated as an adjunct intervention to improve neuromuscular coordination, postural stability, and functional strength. The elastic bands provided low-to-moderate resistance and were applied concurrently during the breathing and balance training phases of the fall prevention exercise program to facilitate controlled muscle activation and joint mobility.

Previous studies have reported that resistance exercises using elastic bands may enhance lower-limb strength, postural stability, and functional mobility in older adults [25,42,43]. Based on this evidence, the elastic band component in the present study was designed to provide proprioceptive input and support coordinated activation of the trunk and lower-limb musculature during movement tasks. Exercise intensity was progressively adjusted by modifying band tension and movement amplitude according to each participant’s functional capacity.

### 2.5. Outcome Measurements

#### 2.5.1. Balancia Program—Static Balance

Participants’ static balance ability was assessed using the Balancia program. Balancia is a software system designed to measure the center-of-gravity (postural sway) and center-of-pressure displacement in conjunction with a Wii Balance Board. This tool has demonstrated high reliability and validity in evaluating static balance [44]. Measurements were conducted under two conditions: with eyes open, in which participants were instructed to gaze at a 15 cm target positioned 3 m ahead for 30 s, and with eyes closed, during which measurements were taken for an additional 30 s [45].

#### 2.5.2. Timed up and Go—Dynamic Balance

The TUG test was used to assess the participants’ dynamic balance ability. The TUG test is a reliable and valid measure used to indirectly evaluate functional mobility and fall risk in daily life [46]. The test was administered according to the following procedures. The participants began seated in a standard chair with armrests (seat height = approximately 46 cm). Upon the examiner’s signal, each participant was instructed to stand, walk 3 m, turn around, return to the chair, and sit down. The total time required to complete the task was measured in seconds. Participants who normally used walking aids were permitted to use their usual assistive devices during testing. After one practice trial, a single test trial was performed, and the completion time was recorded in seconds. The participants were instructed to walk at their usual comfortable pace, and the examiner remained nearby to ensure safety throughout the assessment.

#### 2.5.3. Five Times Sit to Stand Test—Lower-Extremity Muscle Strength

The FTSST was used to assess participants’ lower-extremity functional strength. This test measures the time required to rise from a seated position and sit back down five consecutive times. The FTSST is a reliable measure of the functional capacity of the lower-limb muscles and has been shown to predict fall recurrence and level of independence in performing activities of daily living [47]. During the assessment, the participants were instructed to stand and sit five times as quickly as possible. The timing commenced upon the examiner’s verbal start cue.

#### 2.5.4. Falls Efficacy Scale—Korean Version: Fall Efficacy Assessment

The Falls Efficacy Scale—Korea (FES-K) was used to evaluate the participants’ fear of falling and fall-related self-efficacy during activities of daily living. The FES-K was developed through the translation and adaptation of the Falls Efficacy Scale—International (FES-I) and comprises 10 items [48]. Participants used a 4-point Likert scale ranging from 1 (“very concerned”) to 4 (“not concerned at all”) to rate their confidence in performing common daily activities (e.g., sitting on a chair, bathing, and walking indoors) without falling.

#### 2.5.5. Statistical Analysis

All statistical analyses were performed using IBM SPSS Statistics for Windows (version 19.0; IBM Corp., Armonk, NY, USA). For the general characteristics of the experimental and control groups, a Mann–Whitney U test was used to analyze continuous variables, and a chi-square test was used to analyze sex distribution. The Shapiro–Wilk test was used to assess the normality of each outcome within each group at a significance level of α = 0.05. Because several outcomes showed significant deviations from normality (*p* < 0.05) for at least one group and/or time point (e.g., eyes-closed balance variable and FES-K), non-parametric tests were applied. Data were analyzed using a per-protocol (completer) approach; participants who withdrew during the intervention period were excluded from the final analysis.

A Mann–Whitney U test was used to verify homogeneity between groups and compare pre- and post-intervention differences, whereas a Wilcoxon signed rank test was used to determine the significance of changes within each group pre- and post-intervention. The level of statistical significance was set at *p* < 0.05. Effect sizes were calculated using the equation *r* = *Z*/√N to determine the magnitude of the intervention effect, where 0.1, 0.3, and 0.5 represent small, medium, and large effects, respectively. Additionally, 95% confidence intervals (CIs) for the difference in medians between groups were estimated using the Hodges–Lehmann estimator to provide a measure of precision for the treatment effect.

## 3. Results

### 3.1. General Participant Characteristics

In this study, 26 participants who met the inclusion criteria were randomly assigned to either the experimental (c-tDCS; n = 13) or control (n = 13) group. During the intervention period, two participants in the control group withdrew from the study.

The general characteristics of the groups were compared by conducting a Mann–Whitney U test for continuous variables and a chi-square test for categorical variables (sex). The median age of the experimental group was 74.00 (IQR 10.00) years, while that of the control group was 71.00 (IQR 10.00) years. The median height was 156.00 (IQR 13.00) cm in the experimental group and 156.00 (IQR 7.00) cm in the control group. The median body weights were 54.00 (IQR 14.00) kg and 60.00 (IQR 6.50) kg, respectively. Statistical analysis revealed no significant differences between groups in terms of age, height, weight, or sex distribution.

### 3.2. Changes in Static Balance (Vel Avg) Before and After the Experiment

Assessing static balance ability using Balancia showed no statistically significant differences between the pre- and post-intervention measurements in either group under both eyes-open and eyes-closed conditions (*p* > 0.05).

Under the eyes-open condition, the median postural sway velocity (Velavg) in the experimental group changed from 2.98 (1.08) cm/s to 3.10 (0.86) cm/s (*Z* = −0.31, *p* = 0.754), and that in the control group changed from 3.11 (0.52) cm/s to 2.87 (0.52) cm/s (*Z* = −0.27, *p* = 0.790) (Table 1).

Under the eyes-closed condition, the median postural sway velocity in the experimental group changed from 3.54 (0.65) cm/s to 3.51 (0.87) cm/s (*Z* = −0.04, *p* = 0.972), whereas that in the control group changed from 3.42 (0.79) cm/s to 3.72 (1.01) cm/s (*Z* = −1.78, *p* = 0.075) (Table 1).

A comparison of the change scores between groups using the Mann–Whitney U test revealed no significant differences in any of the measured parameters (*p* > 0.05) (Table 1).

### 3.3. Changes in TUG Before and After the Experiment

In the experimental group, the median TUG performance time significantly decreased from 7.36 (2.04) seconds pre-intervention to 5.56 (1.44) seconds post-intervention (*Z* = −3.18, *p* < 0.001). The control group also showed a statistically significant improvement, with TUG decreasing from 7.19 (2.28) seconds to 6.50 (1.38) seconds (*Z* = −2.05, *p* = 0.041) (Table 1). However, a comparison of the change scores between the two groups using the Mann–Whitney U test revealed that the experimental group achieved significantly greater improvements in dynamic balance than the control group (*U* = 24.00, *p* = 0.005) (Table 1).

### 3.4. Changes in FTSST Before and After the Experiment

The experimental group demonstrated a significant reduction in FTSST performance time, with the median value changing from 9.22 (4.41) seconds pre-intervention to 7.59 (1.72) seconds post-intervention (*Z* = −2.69, *p* = 0.005) (Table 1). In contrast, the control group showed no statistically significant change, with the median time moving from 10.22 (2.09) seconds to 9.63 (1.22) seconds (*Z* = −0.89, *p* = 0.413). When comparing the amount of change between the groups, the experimental group showed significantly greater improvement in lower-extremity functional strength than the control group (*U* = 35.00, *p* = 0.035) (Table 1).

### 3.5. Changes in FES-K After the Experiment

Regarding fall-related self-efficacy, no statistically significant changes were observed in either group. In the experimental group, the median FES-K score remained at 100.00 (1.50) pre-intervention and 100.00 (0.50) post-intervention (*Z* = −0.54, *p* = 0.750). Similarly, the control group’s median score remained unchanged, at 100.00 (0.00) pre-intervention and 100.00 (1.00) post-intervention (*Z* = −0.45, *p* = 1.000). A comparison of the change values between the two groups also revealed no significant difference (*U* = 66.50, *p* = 0.776) (Table 1). The comprehensive data for all outcome measures, including physical functions and fall efficacy, are summarized in Table 1.

## 4. Discussion

This study was designed to contribute to fall prevention efforts in older adults. Participants were divided into an experimental group, who took part in a c-tDCS-based fall prevention exercise program for four weeks, and a control group, who performed the fall prevention exercise program without c-tDCS. This study aimed to evaluate the effects of c-tDCS on physical function and fall efficacy in older adults, thereby providing evidence to support its potential role in fall prevention.

In this study, in the static balance analysis conducted using Balancia, no significant differences were observed between the experimental and control groups in either the eyes-open or eyes-closed condition (*p* > 0.05). The discrepancy between dynamic and static balance improvements may be attributed to the functional specialization of the cerebellum. The cerebellum primarily functions as a neural comparator that performs ‘online’ error correction by integrating vestibular and proprioceptive feedback during active movements like walking and turning [49]. Therefore, the 4-week intervention might have been more effective for dynamic tasks requiring rapid postural adjustments, whereas static balance may require a longer period of stimulation to show measurable changes through cerebellar–cortical modulation [50]. These findings suggest that a short-term, four-week intervention may not have been sufficient to induce measurable changes in the neuroplasticity of the sensory integration necessary for static postural control. Parsaee et al. [33] reported improvements in static balance following a six-week c-tDCS intervention, whereas shorter intervention durations (≤four weeks) showed limited effects. Similarly, Guo et al. [18] noted that single-session or short-term tDCS interventions rarely produce statistically significant changes in center-of-pressure parameters. These results indicate that short-term single-region stimulation, such as tDCS, is unlikely to substantially influence the complex sensory integration mechanisms required for static balance control. Considering that static postural stability depends on the coordinated interaction of the vestibular, visual, and proprioceptive systems, a longer intervention period or multimodal approach is likely necessary to elicit significant improvements.

In this study, the experimental group demonstrated a significant reduction in TUG performance time, from 7.42 s before the intervention to 5.56 s after (*p* = 0.001), indicating a marked improvement compared with the control group (*p* = 0.006). These findings reflect the participants’ enhanced dynamic balance and functional mobility.

A meta-analysis by Guo et al. [18] reported that anodal cerebellar stimulation significantly improves gait stability and balance in tDCS studies involving older adults. Similarly, Khanmohammadi [21] found that high-definition c-tDCS reduces stride variability and enhances gait stability during dual-task walking. Parsaee et al. [33] reported that c-tDCS improves both static and dynamic balance abilities in sedentary older adults. Consistent with these previous findings, this study demonstrated significant improvements in dynamic balance following the c-tDCS intervention.

These outcomes suggest that anodal c-tDCS may enhance the adaptive feedback control of body center movements during gait, promoting faster and more efficient balance recovery responses. This effect is likely mediated by the physiological role of the cerebellum in integrating sensory input from the vestibular system and spinocerebellum and transmitting refined motor control signals to the primary motor cortex (M1).

Furthermore, the fall prevention exercise program used in this study, based on the OEP, incorporated lower-extremity strengthening and postural transition training. As Campbell et al. [22] and Sherrington et al. [23] reported, such exercises facilitate complex improvements in sensory integration and reaction speed. It is noteworthy that the control group also exhibited significant improvements in dynamic balance (TUG). This finding is likely attributable to the efficacy of the OEP-based exercise program itself, which is widely recognized for enhancing functional mobility [22,23]. However, given the nature of the study, the potential influence of a learning effect from repeated measurements or a placebo effect associated with the sham stimulation and interaction with researchers cannot be entirely ruled out. Nevertheless, the significantly greater improvement observed in the experimental group highlights that the neuroplasticity-enhancing effects of c-tDCS amplified the training benefits, offering a synergistic advantage over exercise alone.

In this study, no statistically significant changes were observed in the static balance index (Velavg), whereas dynamic balance (TUG) showed significant improvements. These findings may be attributed to the task-specific characteristics of the OEP, which formed the basis of the fall prevention exercise program, and the mechanistic plausibility of c-tDCS. According to Campbell et al. and Sherrington et al. [22,51], the OEP is designed to emphasize dynamic functional tasks such as walking, weight shifting, and postural transitions, in addition to muscle strengthening exercises. Therefore, it may be more effective in enhancing functional mobility and dynamic postural control than in improving static balance. Furthermore, neurophysiological evidence indicates that c-tDCS facilitates dynamic stability and locomotor adaptation by strengthening error-based motor learning, a process through which motor commands are recalibrated in response to sensory error signals during movement execution [31]. Collectively, these mechanisms support the observed improvement in dynamic balance after c-tDCS combined with OEP-based training.

In this study, the experimental group demonstrated a significant reduction in FTSST performance time in the post-test compared to the pre-test (*p* < 0.05). However, since this study did not assess morphological changes in muscle tissue, these improvements should be attributed to enhanced neural control rather than muscle hypertrophy. Given that the cerebellum is primarily responsible for motor coordination and error correction, c-tDCS likely modulated the excitability of the dentato-thalamo-cortical (DTC) pathway [32]. This facilitation suggests that the intervention optimized neuromuscular efficiency and refined postural sequencing during the sit-to-stand movement, enabling faster task completion without structural changes. This heightened excitability facilitates more efficient motor unit recruitment and adaptive postural control during repetitive, task-oriented exercises like the OEP [34]. Consequently, these neural adaptations likely underlie the observed improvements in lower-extremity muscle strength, contributing to enhanced functional mobility in older adults. These results are consistent with those of Yosephi et al. [19], who reported that “anodal tDCS enhances the effects of postural training by improving lower-extremity muscle strength and postural stability”. Furthermore, similar to the findings of Hernandez-Martinez et al. [25] and Gargallo et al. [26], the present study employed elastic band exercises in conjunction with a fall prevention exercise program, resulting in significant improvements in lower-extremity muscle strength among older adults, thereby supporting the outcomes of previous research.

The mechanism underlying the improvement observed in this study may be attributable to the ability of tDCS to enhance the excitability of the cerebello-thalamo-cortical pathway, thereby lowering the activation threshold of motor units and improving coordination efficiency during muscle contraction [31]. This increased excitability facilitates the recruitment of more motor units at the same exercise intensity, thereby enhancing neuromuscular efficiency. These findings suggest that the improvement in FTSST performance observed in the experimental group may be explained by the synergistic interaction between the neuroplasticity-enhancing effects of c-tDCS and muscle activation effects of elastic resistance exercise.

In this study, FES-K scores, which were used to assess the participants’ confidence in avoiding falls, showed no statistically significant change (*p* > 0.05). This finding suggests that improvements in physical performance do not necessarily translate into increased self-confidence or reduced psychological fear of falling. Huh et al. [48] reported that FES-K scores are influenced more by cognitive and emotional factors, such as self-efficacy and anxiety levels, than by the actual experience of falls. Similarly, Clemson et al. [52] observed significant improvements in fall efficacy only when behavioral modification was combined with balance and strength training for 12 weeks or longer. The synergy between c-tDCS and the OEP appears to be a key factor, as c-tDCS induces a state of increased neuroplasticity that allows for better internalization of motor patterns [29,30]. Furthermore, the reduction in TUG time in the experimental group is clinically meaningful. Previous research indicates that such improvements are strong predictors of reduced fall risk in community-dwelling older adults, suggesting that our intervention provides practical benefits beyond statistical significance [53].

However, despite these physical improvements, several critical limitations must be acknowledged to properly contextualize these findings. First, the results should be interpreted as preliminary given the pilot nature of the study. The combination of a relatively small sample size (*n* = 26) and a short four-week duration increase the risk of both Type I (false positive) and Type II (false negative) errors. Second, regarding sample characteristics, the included participants were limited to healthy, community-dwelling older adults without a history of falls. This precludes the generalization of our findings to higher-risk clinical populations where fall prevention is most urgent. Furthermore, the analysis was conducted using a per-protocol approach, excluding data from two participants who dropped out. While this ensures that the results reflect the effects of the completed intervention, the exclusion of missing data may have introduced attrition bias compared to an intention-to-treat analysis. Third, the measurement protocol for functional outcomes (TUG and FTSST) involved a single trial following one practice attempt. This deviates from best-practice protocols that recommend averaging multiple trials to enhance reliability, which may have introduced measurement error and potential bias. Additionally, the study did not include long-term follow-up or prospective clinical trial registration. Finally, the sham protocol employed in this study (30 s of stimulation) is a widely accepted method for blinding in tDCS research [41,54]. However, we did not perform a post-experiment blinding success check; therefore, the integrity of blinding could not be quantitatively verified, which remains a potential threat to internal validity. Future studies incorporating larger cohorts, extended intervention durations, and objective neurophysiological measures are needed to further validate and expand upon these findings.

## 5. Conclusions

The results of this pilot study suggest that the c-tDCS-based fall prevention exercise program may effectively improve lower-extremity muscle strength and dynamic balance in older adults. These findings suggest that c-tDCS enhances motor performance by promoting neuroplasticity and improving motor control efficiency. However, no significant improvements were observed in static balance or fall efficacy, indicating that short-term interventions may have a limited influence on sensory integration and psychological factors.

This study was limited by its small sample size, short four-week intervention period, and reliance on subjective questionnaire-based assessments. Therefore, future studies should include larger sample populations, extended intervention durations, and objective neurophysiological measures to further validate and expand upon these findings.

## Figures and Tables

**Table 1:** Comparison of physical functions and fall efficacy between the c-tDCS group and the control group before and after the intervention.

Variable	Group	c-tDCS Group Median (IQR)	Control Group Median (IQR)	95% CI for Median Diff. ^†^	*U* (*p*)	Effect Size (*r*)
EO velocity (cm/s)	Pre	2.98 (1.08)	3.11 (0.52)			
	Post	3.10 (0.86)	2.87 (0.52)			
	Pre-post	−0.06 (0.49)	0.01 (0.41)	−0.23 to 0.32	68.00 (0.87)	0.03
	*Z* (*p*)	−0.31 (0.75)	0.79 (0.79)			0.06/0.05
EC velocity (cm/s)	Pre	3.54 (0.65)	3.42 (0.79)			
	Post	3.51 (0.87)	3.72 (1.01)			
	Pre-post	−0.07 (0.75)	0.15 (0.36)	−0.62 to 0.19	52.00 (0.28)	0.21
	*Z* (*p*)	−0.04 (0.97)	−1.78 (0.08)			0.01/0.35
TUG (s)	Pre	7.36 (2.04)	7.19 (2.28)			
	Post	5.56 (1.44)	6.50 (1.38)			
	Pre-post	−1.95 (1.80)	−0.47 (1.37)	−2.13 to −0.46	24.00 (0.01 *)	0.51
	*Z* (*p*)	−3.18 (0.00 *)	−2.05 (0.04 *)			0.62/0.40
FTSST (s)	Pre	9.22 (4.41)	10.22 (2.09)			
	Post	7.59 (1.72)	9.63 (1.22)			
	Pre-post	−1.78 (4.20)	−0.44 (2.18)	−3.52 to –0.05	35.00 (0.04 *)	0.41
	*Z* (*p*)	−2.69 (0.01 *)	−0.89 (0.41)			0.53/0.17
FES-K	Pre	100.00 (1.50)	100.00 (0.00)			
	Post	100.00 (0.50)	100.00 (1.00)			
	Pre-post	0.00 (0.00)	0.00 (0.00)	0.00 to 0.00	66.50 (0.78)	0.06
	*Z* (*p*)	−0.54 (0.75)	−0.45 (1.00)			0.11/0.09

Values are presented as median (interquartile range). *Z*: Wilcoxon signed-rank test; *U*: Mann–Whitney U test; *r*: Effect size; * *p* < 0.05. In the ‘Effect Size (*r*)’ column, *Z* (*p*) row values are presented as ‘c-tDCS/Control’; c-tDCS: cerebellar transcranial direct-current stimulation; EO: eyes open; EC: eyes closed; STS: Sit to stand; TUG: Time Up to Go; FES-K: Falls Efficacy Scale—Korean, ^†^ 95% confidence interval for the median difference between groups (Hodges–Lehmann estimator).

## Data Availability

The datasets generated during the current study are not publicly available due to participant privacy and ethical restrictions but are available from the corresponding author upon reasonable request.

## Abbreviations

tDCS	Transcranial direct-current stimulation
C-TDCS	cerebellum transcranial direct-current stimulation
OEP	Otago Exercise Program
TUG	Time Up to Go
FTSST	Five Times Sit to Stand Test
EO	Eyes open
EC	Eyes closed
FES-K	Falls Efficacy Scale—Korean
